# Progressive and Prognostic Performance of an Extracellular Matrix-Receptor Interaction Signature in Gastric Cancer

**DOI:** 10.1155/2020/8816070

**Published:** 2020-10-29

**Authors:** Xiangchou Yang, Liping Chen, Yuting Mao, Zijing Hu, Muqing He

**Affiliations:** ^1^Department of Hematology and Medical Oncology, The Second Affiliated Hospital and Yuying Children's Hospital of Wenzhou Medical University, Wenzhou, 325000 Zhejiang Province, China; ^2^Department of Pharmacy, Sir Run Run Shaw Hospital, School of Medicine, Zhejiang University, Hangzhou, 310000 Zhejiang Province, China; ^3^Second Clinical College of Medicine, Wenzhou Medical University, Wenzhou, 325000 Zhejiang Province, China; ^4^College of Pharmaceutical Sciences, Wenzhou Medical University, Wenzhou, 325000 Zhejiang Province, China

## Abstract

The role of an extracellular matrix- (ECM-) receptor interaction signature has not been fully clarified in gastric cancer. This study performed comprehensive analyses on the differentially expressed ECM-related genes, clinicopathologic features, and prognostic application in gastric cancer. The differentially expressed genes between tumorous and matched normal tissues in The Cancer Genome Atlas (TCGA) and validation cohorts were identified by a paired *t*-test. Consensus clusters were built to find the correlation between clinicopathologic features and subclusters. Then, the least absolute shrinkage and selection operator (lasso) method was used to construct a risk score model. Correlation analyses were made to reveal the relation between risk score-stratified subgroups and clinicopathologic features or significant signatures. In TCGA (26 pairs) and validation cohort (134 pairs), 25 ECM-related genes were significantly highly expressed and 11 genes were downexpressed in gastric cancer. ECM-based subclusters were slightly related to clinicopathologic features. We constructed a risk score model = 0.081∗log_2_ (CD36) + 0.043∗log_2_ (COL5A2) + 0.001∗log_2_ (ITGB5) + 0.039∗log_2_ (SDC2) + 0.135∗log_2_ (SV2B) + 0.012∗log_2_ (THBS1) + 0.068∗log_2_ (VTN) + 0.023∗log_2_ (VWF). The risk score model could well predict the outcome of patients with gastric cancer in both training (*n* = 351, HR: 1.807, 95% CI: 1.292-2.528, *P* = 0.00046) and validation (*n* = 300, HR: 1.866, 95% CI: 1.347-2.584, *P* = 0.00014) cohorts. Besides, risk score-based subgroups were associated with angiogenesis, cell adhesion molecules, complement and coagulation cascades, TGF-beta signaling, and mismatch repair-relevant signatures (*P* < 0.0001). By univariate (1.845, 95% CI: 1.382-2.462, *P* < 0.001) and multivariate (1.756, 95% CI: 1.284-2.402, *P* < 0.001) analyses, we regarded the risk score as an independent risk factor in gastric cancer. Our findings revealed that ECM compositions became accomplices in the tumorigenesis, progression, and poor survival of gastric cancer.

## 1. Introduction

As a common tumor of the digestive system, gastric cancer is the fifth common malignant tumor and the third leading cause of cancer death in the world [1, 2]. Due to the occult course of gastric cancer, it is of great significance to clarify the pathogenesis and find effective markers for gastric cancer.

In recent years, studies have shown that the extracellular matrix (ECM) remodeling, namely, the synthesis, distribution, and degradation of ECM, is closely connected to the differentiation, proliferation, invasion, and metastasis of malignant tumors [3]. ECM constitutes the main part of the extracellular microenvironment [4]. It is a complex organic unity constructed by a variety of insoluble extracellular macromolecules in a certain proportion and structure. It is the site of cell survival and activity, with physical functions such as connection, support, water retention, pressure resistance, and protection. In addition, by integrin or other cell surface receptors, it can directly interact with cells to regulate growth, metabolism, function, migration, proliferation, and differentiation of cells, thus to adjust functions of the whole tissue and organs [4]. Recent studies on solid tumors such as breast cancer and ovarian cancer have suggested that ECM underwent a remodeling process similar to embryonic development in tumor progression. The reconstructed ECM then forms a loose microenvironment for cancer cells, giving rise to high proliferation, low differentiation, and invasion and metastasis of tumor cells [5]. Therefore, the identification of prominent ECM-relevant tumor markers that derive the biological perspective into the development and progression of gastric cancer would be of clinical value. In this study, the differentially expressed ECM-relevant markers were identified between gastric cancer and normal tissues. Based on the selection operator (lasso) regression model, it revealed that the ECM-relevant markers exhibited a great value to predict the prognosis of gastric cancer.

## 2. Methods

### 2.1. Datasets

We downloaded The Cancer Genome Atlas-Stomach Adenocarcinoma (TCGA-STAD) data from the UCSC Xena browser (https://xena.ucsc.edu/) [6]. The RNA-sequencing data were unified into log_2_ (FPKM + 1) (fragments per kilobase million (FPKM)). The validation data GSE29272 [7] and GSE62254 [8] were downloaded from Gene Expression Omnibus datasets (https://www.ncbi.nlm.nih.gov/geo/).

### 2.2. Genes of Researched Signatures

We investigated all ECM-receptor interaction-related genes (KEGG hsa04512) from the Kyoto Encyclopedia of Genes and Genomes (KEGG) (https://www.kegg.jp/) [9]. Besides, genes of cell adhesion molecules (CAMs) (KEGG hsa04514), complement and coagulation cascades (KEGG hsa04610), TGF-beta signaling pathway (KEGG hsa04530), base excision repair (KEGG hsa03410), DNA replication (KEGG hsa03030), nucleotide excision repair (KEGG hsa03420), and mismatch repair (KEGG hsa03430) were also identified from KEGG (Table 1).

### 2.3. Building a lasso Regression Model

We conducted the univariate analysis of each ECM-receptor interaction-related genes. Then, the genes with *P* < 0.05 were selected in the establishment of a lasso regression model. The lasso regression model was built by the package “glmnet” of R [10]. According to the lasso model, each patient is assigned a risk score. We defined patients with a risk score ≥ median value in the high-risk group (*N* = 175); otherwise, in the low-risk group (*N* = 176).

### 2.4. Statistical Analyses

We identified differentially expressed genes between tumorous and matched normal tissues in TCGA and validation cohorts by a paired *t*-test. Consensus clusters were built by the package “ConsensusClusterPlus” of R [11]. We identified a consensus matrix of TCGA for *k* from 2 to 9. Gene set enrichment analysis (GSEA) was used to analyze the most enriched gene sets of the high- and low-risk groups [12, 13]. Packages “clusterProfiler” [14], “org.Hs.eg.db,” “enrichplot,” and “GO plot” [15] of R were applied to perform GO analyses and visualize the results. The package “GSVA” was applied to get single-sample gene set enrichment analysis (ssGSEA) of relevant signatures [16]. The package “survminer” was used to visualize the survival time of high- and low-risk groups. A *P* value > 0.05 was considered to indicate a statistically significant difference. All analyses were conducted with R (https://www.r-project.org/). The hazard ratios were shown with 95% confidence interval (95% CI).

## 3. Results

### 3.1. Differentially Expressed Genes of an ECM-Receptor Interaction Signature

**Figure 1:**
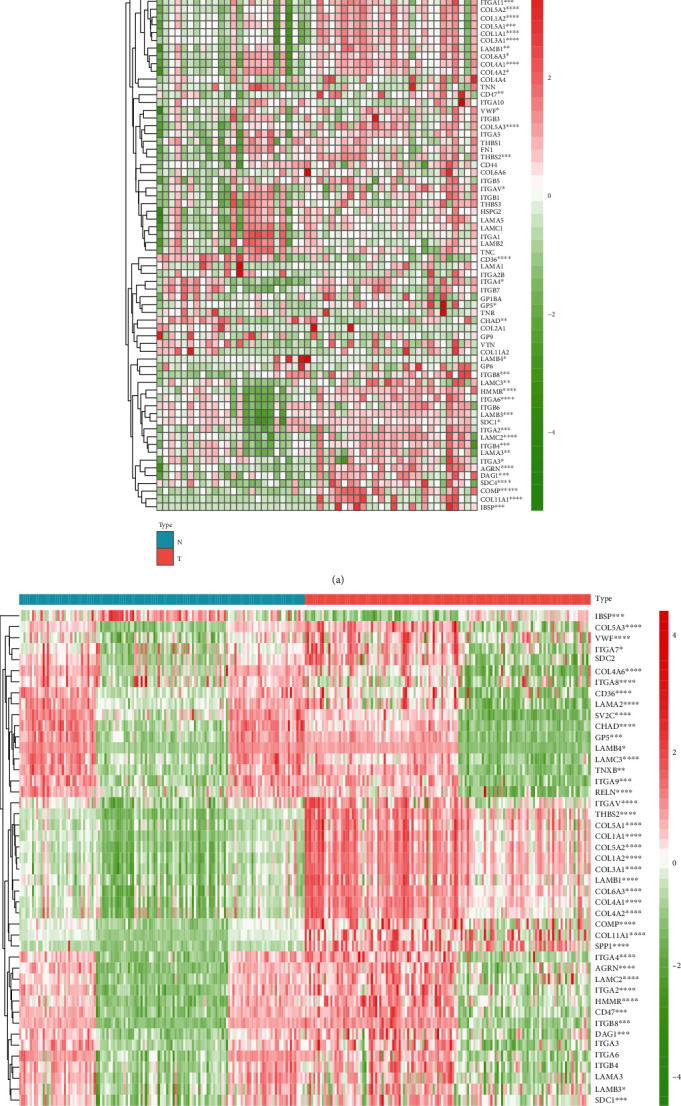
Differential expression of ECM-receptor interaction-related genes between tumor and matched normal tissues in TCGA and validation cohorts of gastric cancer. (a) The heat map showed differential expression of all ECM-receptor interaction-related genes of 26 pairs of tumorous and matched normal tissues of gastric cancer in TCGA. (b) The heat map validated 45 ECM-receptor interaction-related genes that were identified in the validation cohort (GSE29272) with 134 pairs of tumorous and matched normal tissues. TCGA: The Cancer Genome Atlas; N: adjacent tissue to cancer; T: tumorous tissue. ^∗^*P* < 0.05, ^∗∗^*P* < 0.01, ^∗∗∗^*P* < 0.001, and ^∗∗∗∗^*P* < 0.0001.

In TCGA cohort, there were 26 pairs of tumorous and matched normal tissues enrolled in the study. As shown in Figure 1(a) and Supplementary Figure 1, the expressions of 46 ECM-receptor interaction-related genes were significantly different in contrast to adjacent tissues. In an independent cohort with 134 pairs of tumorous and matched normal tissues, 36 genes had the obvious uniformity with the expression changes. The expressions of *AGRN*, *CD47*, *COL11A1*, *COL1A2*, *COL3A1*, *COL4A1*, *COL4A2*, *COL5A1*, *COL5A2*, *COL5A3*, *COL6A3*, *COMP*, *DAG1*, *HMMR*, *ITGA2*, *ITGA4*, *ITGAV*, *ITGB8*, *LAMB1*, *LAMB3*, *LAMC2*, *SPP1*, *THBS2*, *VWF*, and *SDC1* were significantly highly expressed in gastric cancer, while 11 genes *CD36*, *CHAD*, *COL4A6*, *ITGA8*, *ITGA9*, *LAMA2*, *RELN*, *SV2C*, *TNXB*, *LAMB4*, and *LAMC3* were downexpressed in tumorous tissues (Figure 1(b) and Supplementary Figure 2).

### 3.2. Building Consensus Clusters and Correlation between Clinicopathologic Features and Clusters

**Figure 2:**
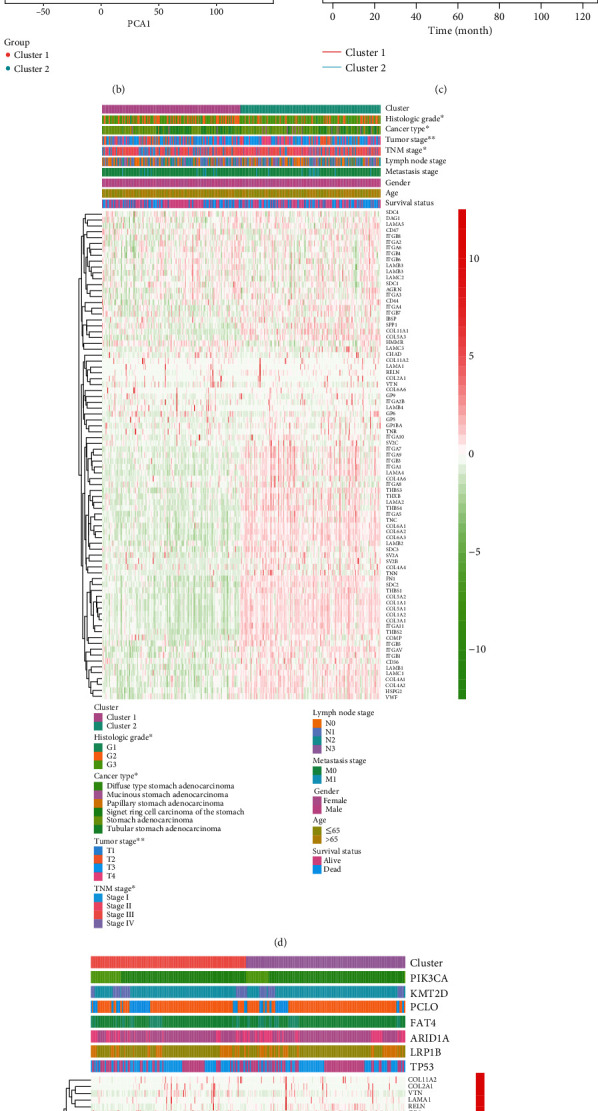
Identification of subclusters stratified by ECM-receptor interaction-related genes and correlation between subclusters and clinicopathologic features. (a) Identification of the consensus matrix of TCGA cohort for *k* = 2. (b) Principal component analysis of subclusters. (c) Survival curve of subclusters stratified by ECM-receptor interaction-related genes. (d, e) Correlation analyses between tumor characteristics or mutations and the subclusters. ^∗^*P* < 0.05, ^∗∗^*P* < 0.01.

We identified consensus matrixes of TCGA for *k* from 2 to 9 (Figure 2(a) and Supplementary Figure 3). In consideration of discrimination and simplicity, we chose *k* = 2 to build consensus clusters. Principal component analysis (PCA) showed that two consensus clusters had a certain degree of differentiation (Figure 2(b)). Patients in cluster 2 (*N* = 190) had worse outcomes than patients in cluster 1 (*N* = 185) (*P* = 0.0032) (Figure 2(c)). Besides, stratified clusters were slightly related to the histologic grade, cancer type, tumor stage, and TNM stage, while presenting no correlation with *PIK3CA*, *KMT2D*, *PCLO*, *FAT4*, *ARID1A*, *LRP1B*, and *TP53* mutations (Figures 2(d)–2(f) and Supplementary Table 1).

### 3.3. Establishment of the lasso Regression Model

**Figure 3:**
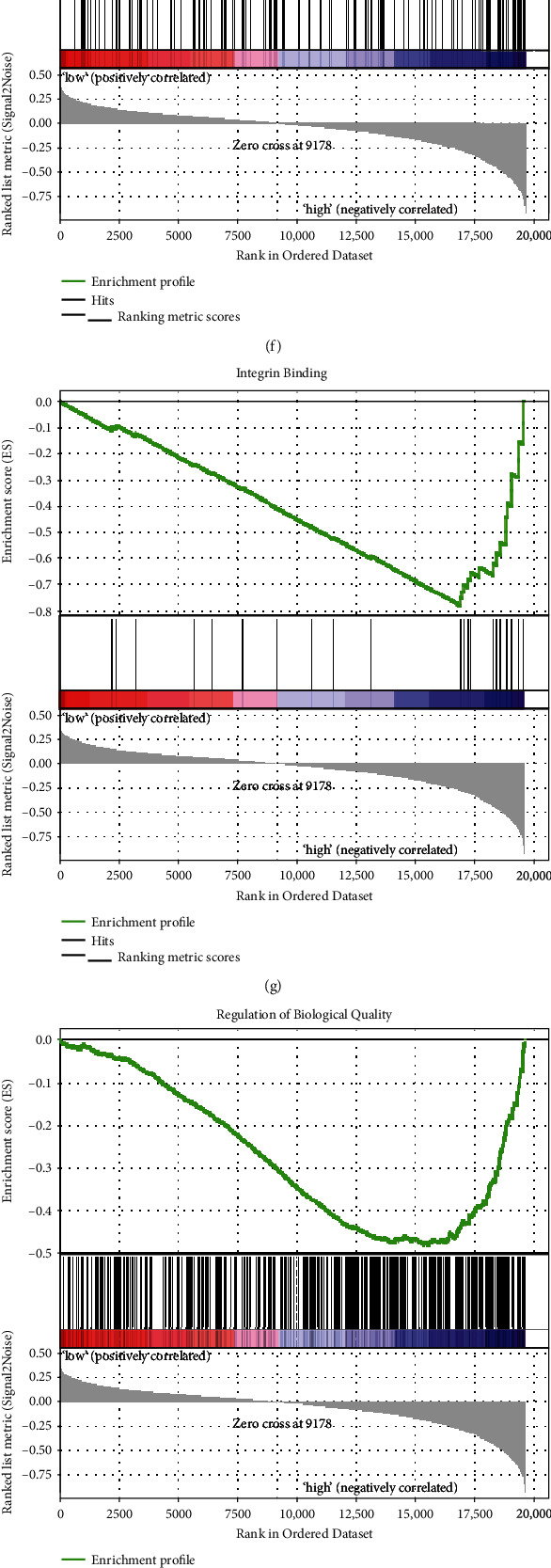
Construction of the lasso regression model with ECM-receptor interaction-related genes and enrichment analyses. (a) The relation between partial likelihood deviances and number of genes involved in the risk model. (b) The solution paths of the risk model. (c) The coefficients of each gene involved in the risk model. (d–h) Top five enriched gene sets between high- and low-risk groups identified by the risk model. (i) The GO analysis between high- and low-risk groups identified by the risk model. (j) Top genes refer to the top BP. GO: BP: biological process; MF: molecular function; CC: cellular component; logFC: log_2_(fold change).

To better predict the outcome of gastric cancer patients, we calculated the hazard ratio with 95% confidence interval of all ECM-receptor interaction-related genes and 25 of them with *P* < 0.05, which were enrolled in the establishment of the lasso regression model (Table 2). Figures 3(a) and 3(b) show the solution paths and partial likelihood deviances of the building process of the lasso regression model. The risk score model = 0.081∗log_2_ (CD36) + 0.043∗log_2_ (COL5A2) + 0.001∗log_2_ (ITGB5) + 0.039∗log_2_ (SDC2) + 0.135∗log_2_ (SV2B) + 0.012∗log_2_ (THBS1) + 0.068∗log_2_ (VTN) + 0.023∗log_2_ (VWF) (Figure 3(c)). GSEA showed the top enriched gene sets: protein complex binding, GTPase activity, organ morphogenesis, integrin binding, and regulation of biological quality (Figures 3(d)–3(h)). GO analyses revealed the top enriched biological process (BP), molecular function (MF), and cellular component (CC) (Figure 3(i)). The circular plot showed that 17 genes were highly related to the GO term (Figure 3(j)).

### 3.4. Predictive Ability of the Risk Model

**Figure 4:**
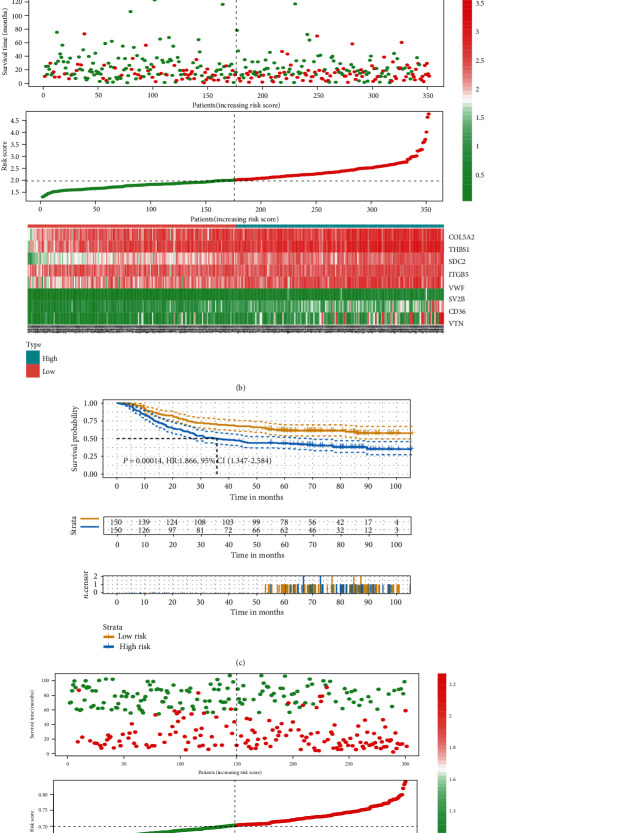
Survival analyses and distribution of the risk model in the training and validation cohorts. (a) Survival curve of the high- and low-risk groups identified by the risk model in TCGA cohort. (b) The distribution of survival month, risk score, and gene expression in TCGA cohort. (c) Survival curve of the high- and low-risk groups identified by the risk model in the validation cohort (GSE62254). (d) The distribution of survival month, risk score, and gene expressions in the validation cohort (GSE62254).

In TCGA cohort (*n* = 351), the risk model could predict the outcome of patients with gastric cancer (HR: 1.807, 95% CI: 1.292-2.528, *P* = 0.00046), whose reliability and credibility were stronger than those of the consensus clusters (*P* = 0.032) (Figure 4(a)). Besides, in another independent cohort (GSE62254) (*n* = 300), the risk model could still provide excellent prediction accuracy (HR: 1.866, 95% CI: 1.347-2.584, *P* = 0.00014) (Figure 4(c)). The distribution of survival time, risk score, and gene expressions showed that patients in the high-risk group had shorter disease survival time in both TCGA (Figure 4(b)) and validation cohorts (Figure 4(d)).

### 3.5. Correlation between Risk Groups and Clinicopathologic Features

**Figure 5:**
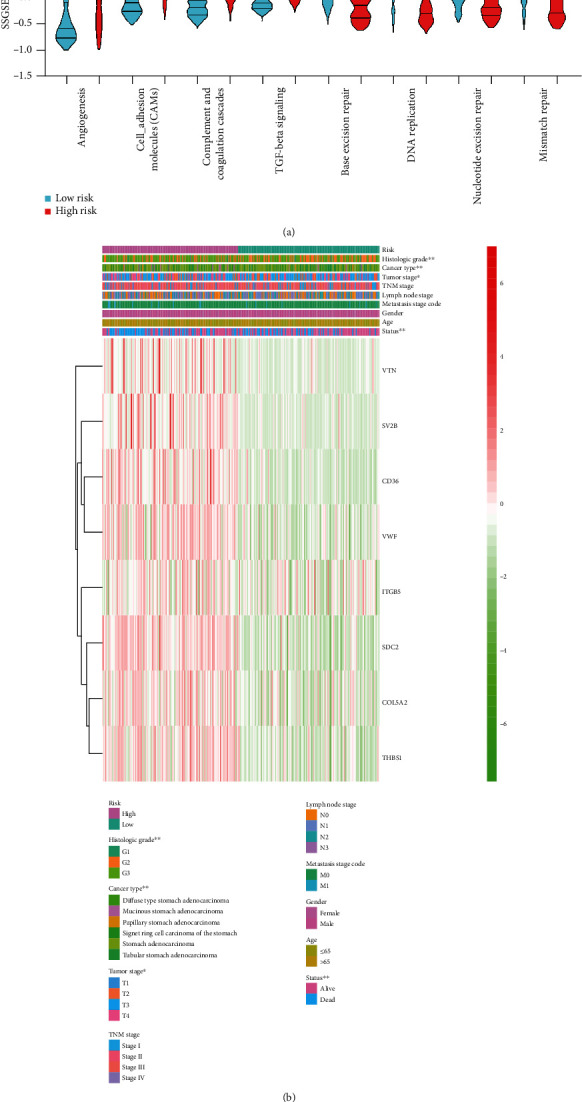
Relevant signatures and clinicopathologic features of the risk groups. (a) The violin plot showed high- and low-risk groups identified by different signatures. Within each group, the middle line represents the mean value of signature genes, and the bottom and top lines represent the 25th and 75th percentiles, respectively. (b) Correlation analyses between tumor characteristics and the risk groups. ^∗∗^*P* < 0.01.

To explore the underlying mechanisms of the risk group, we compared relevant signatures in the high- and low-risk groups. As shown in Figure 5(a), we found the signatures angiogenesis, cell adhesion molecules, complement and coagulation cascades, and TGF-beta signaling enriched in the high-risk group, while mismatch repair-relevant signatures base excision repair, DNA replication, nucleotide excision repair, and mismatch repair were not in the group. Furthermore, the risk stratification was highly correlated with the histologic grade (*P* < 0.01), cancer type (*P* < 0.01), tumor stage (*P* < 0.05), and living status (*P* < 0.01) (Figure 5(b)).

### 3.6. Univariate and Multivariate Analyses of the Risk Score and Clinicopathologic Features

**Figure 6:**
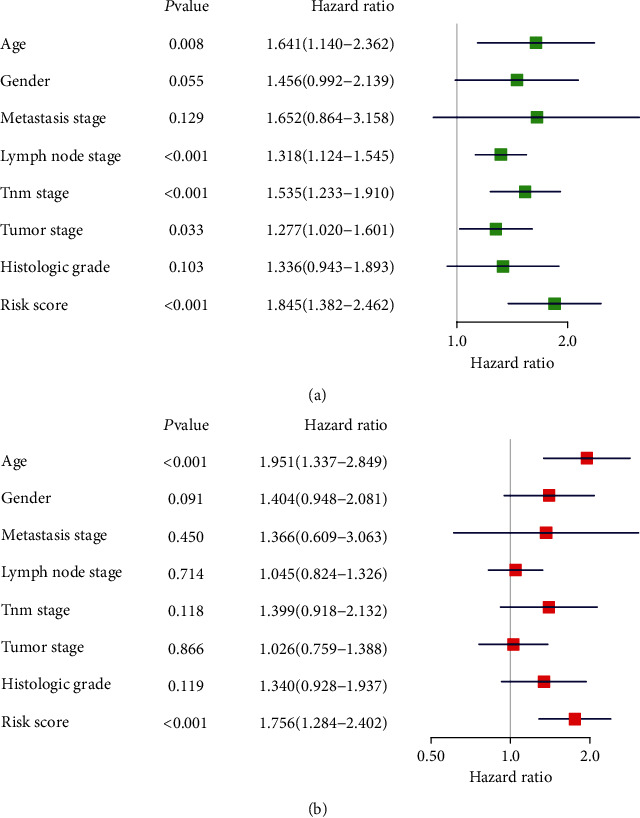
The forest plot of clinicopathologic features and the risk score. (a) Univariate analysis of clinicopathologic features and the risk score in TCGA cohort. (b) Multivariate analysis of clinicopathologic features and the risk score in TCGA cohort. Hazard ratios are shown with 95% confidence interval.

In the univariate analysis, age (1.641, 95% CI: 1.140-2.362, *P* = 0.008), the lymph node stage (1.318, 95% CI: 1.124-1.545, *P* < 0.001), the TNM stage (1.535, 95% CI: 1.233-1.910, *P* < 0.001), the tumor stage (1.277, 95% CI: 1.020-1.601, *P* = 0.033), and the risk score (1.845, 95% CI: 1.382-2.462, *P* < 0.001) were risk factors for gastric cancer (Figure 6(a)). In the multivariate analysis, age (1.951, 95% CI: 1.337-2.849, *P* < 0.001) and the risk score (1.756, 95% CI: 1.284-2.402, *P* < 0.001) were the main risk factors for gastric cancer (Figure 6(b)).

## 4. Discussion

Gastric cancer is characterized by insidious onset, easy metastasis, early misdiagnosis, and high recurrence rate [17]. Due to the lack of a simple domestic screening system, most patients with gastric cancer are in the late stage when first diagnosed, greatly influencing their clinical therapeutic effect and survival quality [18]. Within this context, tumor markers, in the field of biochemistry, have received increasing attention for their characters such as noninvasive, safe, simple, inexpensive, and easy to monitor dynamically [19]. For gastric cancer, many tumor markers have been detected from the perspective of genetic traits or genetic modification. In this study, we revealed that in gastric cancer, many ECM-relevant molecules also were effective tumor markers, possessing an important value in clinical application.

In previous research, ECM-relevant molecules have been identified as progression and prognostic biomarkers in some other solid tumors that were used for impacting clinical decisions and overall outcomes. For example, in colon adenocarcinoma (CAC), *COL1A2*, *THBS2*, and *COL1A1* were related to prognosis [20]. In addition, it was found that the level of *ITGA5* in CAC was significantly linked to overall survival (OS), which might serve as an independent prognostic indicator [21]. In neuroblastoma, it has been revealed that there existed an association between *SDC3* expression and improved prognosis [22]. Additionally, the high expression level of *SDC3* was also associated with poor prognosis in patients with renal cell carcinoma [23]. For lung cancer, *COL5A1* was highly expressed in patients with recurrence and short survival [24]. *SSP1* was upregulated in tumor tissues, and low expression of SSP1 had a significant relationship with the better outcome [25]. Moreover, according to the reported references, *FN1* likely represented a signature biomarker for lung cancer in the prediction of responses to treatments [26]. In contrast to these cancer types that we have discussed, ECM-receptor interaction-relevant genes have been poorly studied as progressive and prognostic biomarkers in gastric cancer. Through the KEGG database, we systematically examined 84 ECM-receptor interaction-relevant genes in this study and found that most of them were differentially expressed in gastric cancer tissues. On the basis of these genes, we divided patients into two subclusters. As we had expected, the subclusters exhibited good prognostic performance (*P* = 0.032). For better prediction of survival with ECM-receptor interaction-relevant genes, lasso regression analysis was then conducted. Thereinto, we found that eight significant genes (*VTN*, *SV2B*, *CD36*, *VWF*, *ITGB5*, *SDC2*, *COL5A2*, and *THBS1*) were related to ECM-receptor interaction and an eight-gene risk score model was constructed based on them. The risk score model had its favorable performance in predicting prognosis of gastric cancer. The eight genes may be potential prognostic markers for gastric cancer.

In a variety of tumors, such as cervix neoplasia [27], ovarian cancer [28], and prostate cancer [29], *VTN* was considered a promising biomarker, which encoded vitronectin, an adhesive glycoprotein that connected cells with ECM. Recently, a report also revealed that *VTN* was a poor prognostic factor in gastric cancer [30]. Likewise, *VWF*, encoding von Willebrand factor that is a platelet adhesion glycoprotein, has been widely used as a biomarker in cancer, and it also has been identified as a new therapeutic target in gastric cancer [31]. As for *THBS1*, encoding thrombospondin 1, it took part in angiogenesis and tumor progression, whose increased expression was significantly correlated with tumor differentiation [32]. *COL5A1*, encoding an alpha chain of type V collagen, was a promising prognostic marker considered to have a good potential for the treatment of patients with gastric cancer as well [33]. The expression of *CD36* was reported in relation to gastric cancer metastasis via O-GlcNAcylation [34]. However, the current literature mostly explores the role of one gene in gastric cancer and rarely links them to explore the combined effect on the gastric cancer treatment. Besides, *ITGB5*, encoding integrin-*β*5, was thought to be involved in the regulation of tumor initiation and progression by mediating links between cells and ECM. The literature reported in glioblastoma [35], hepatocellular carcinoma [36], and cervical cancer [37] that *ITGB5* could serve as a predictive biomarker. In *ITGB5*, the gene expression analysis identified that its expression was elevated in gastric tumor tissue [38]. Nevertheless, the function of *ITGB5* in gastric cancer is not yet fully elucidated. As for *SV2B* and *SDC2*, encoding a member of the synaptic vesicle protein 2 and syndecan 2, respectively, both of them have not been fully studied in gastric cancer. *SV2B* was identified as a key prognosis-associated marker in glioblastoma multiforme and prostate cancer [39, 40]. In spite of this, the study of *SV2B* in tumors is still limited. Relatively speaking, *SDC2* has been well studied in various tumors, especially in colorectal cancer, lung cancer, prostate cancer, and esophageal squamous cell carcinoma [41–45]. According to the discussion above, we considered that *SV2B* and *SDC2* deserved to be further studied in gastric cancer. The disruption in ECM organization lost its regularity, which will compromise gastric cancer foci. ECM compositions became accomplices in the tumorigenesis, progression, and poor survival of gastric cancer. The aberrant ECM signature should be simultaneously inhibited in the treatment of gastric cancer [46].

We further investigated the possible mechanisms underlying the differences between low- and high-risk groups. It was found that there existed a significant difference in angiogenesis between the two groups. As you know, it has been suggested that angiogenesis provided nutrients for tumor growth and pathways for cell metastasis [47]. Consistent with our research, the angiogenesis signature was upregulated in the high-risk group. Besides, the angiogenesis depends on migration and proliferation of vascular endothelial cells [48]. In this process, endothelial cells must attach to each other and to the extracellular matrix to form and expand new microvessels. ECM is one of the critical influencers in the survival of vascular endothelial cells [49]. Thus, we speculated that these differentially expressed genes could promote the formation of tumor blood vessels and further affect the development and prognosis of tumors. Moreover, cell adhesion molecules presented as one of the main media between cells and ECM. The changes of cell adhesion molecules could affect multiple signaling pathways, thereby affecting the pathophysiology of cancer tissues [50]. In addition to possible changes in angiogenesis and cell adhesion molecules, complement and coagulation cascades were also affected in gastric cancer, which might participate in tumor progression and prognosis. Increasing evidence has indicated that complement and coagulation cascades were significantly involved in the signaling pathway in gallbladder cancer [51], clear cell renal cell carcinoma [52], small-cell lung cancer [53], epithelial ovarian cancer [54], bladder cancer [55], and head and neck cancer [56]. In gastric cancer, Gu et al. once pointed that complement and coagulation cascades were significantly enriched pathways [57]. However, the research about it in gastric cancer is insufficient, and there is no direct evidence to clarify that the upregulation of this pathway connects with the prognosis of gastric cancer. From the results in this study, we also found that TGF-*β* signaling pathway is upregulated in gastric cancer, which was in line with the results of existing research. The dysregulated pathway could promote the generation of ECM [58], leading to tissue fibrosis. An overactivated TGF-*β* signaling pathway could induce tumor growth and metastasis by promoting epithelial-mesenchymal transformation and angiogenesis [59]. Of course, the results indicated that we could further research the relationship between the eight significant genes and TGF-*β*.

Furthermore, the downregulation of base excision repair and nucleotide excision repair signatures in the high-risk group was consistent with the current research in gastric cancer. Particularly, DNA mismatch repair is one of the most prevalent pathways involved in a damaged base excision repair system. Absence of base excision repair could result in the accumulation of DNA damage, leading to cancer malignant transformations and poor prognosis. This imbalance was also associated with DNA polymorphism regulation, and such uncorrected false DNA variant likely had relation to cancer risk [60]. The defects in nucleotide excision repair would lead to the increased instability of the genome. Besides, unrepaired DNA damage possibly increased genetic susceptibility to cancers and risk of carcinogenesis [61]. Thus, according to the mentioned results above, the association between the excision repair and eight significant genes deserved to be further explored.

Recent research suggested that the impact of age as an independent risk factor on gastric cancer may differ depending on the cancer stage [62]. Although the finding of age as an independent risk factor in this study had a certain particular value, large-scale clinical data is urgently needed to verify and thus to direct the establishment of a clinical treating scheme. We identified a risk score model to predict prognosis of patients with gastric cancer and validate it in an independent cohort. For the simple and convenient assessment, we could choose it to provide some references. However, we need to acknowledge that the risk score is a relative value, which varies in different institutes and different detection methods. After unifying the testing methods, we need to collect as many samples as possible to identify the cut-off value to guide the oncologists.

## 5. Conclusions

In conclusion, we produced comprehensive analyses to investigate the vital role of an ECM-receptor interaction signature in gastric cancer. ECM compositions became accomplices in the tumorigenesis, progression, and poor survival of gastric cancer.

## Figures and Tables

**Table 1:** Genes of researched signatures.

Gene signature	Gene	Source
ECM-receptor interaction	GP1BA, COL6A2, COL6A3, GP1BB, COL5A2, COL6A1, LAMA1, VWF, HSPG2, TNN, FN1, ITGA9, GP9, COMP, IBSP, CD36, CHAD, GP5, VTN, THBS4, ITGA4, ITGA3, ITGA2B, ITGA7, ITGA5, COL5A1, COL4A6, ITGA11, SV2C, COL2A1, COL3A1, COL4A1, AGRN, COL4A2, COL4A4, ITGB3, ITGB4, RELN, ITGB5, ITGB6, ITGB7, LAMC2, ITGAV, ITGB1, LAMB2, SPP1, LAMB3, LAMC1, COL1A1, LAMA4, LAMA5, LAMB1, COL1A2, ITGA10, GP6, ITGA8, LAMB4, TNR, CD47, SV2A, CD44, DAG1, TNXB, LAMA3, LAMA2, SDC3, ITGB8, ITGA6, ITGA2, ITGA1, SV2B, TNC, COL11A1, LAMC3, COL11A2, HMMR, SDC2, SDC4, COL5A3, THBS3, COL6A6, THBS2, SDC1, THBS1	KEGG hsa04512
Cell adhesion molecules (CAMs)	CDH5, JAM3, CDH3, NLGN3, CDH4, CD80, NLGN1, CD86, CD28, CD274, PDCD1LG2, ITGA9, ITGAL, NRCAM, ITGAM, CD34, CD276, ICOSLG, CADM3, ITGA4, ICOS, SIGLEC1, CADM1, HLA-G, CLDN20, PECAM1, CD22, ITGB7, SELL, VCAM1, ITGAV, SELP, SPN, ITGB1, SELPLG, ITGB2, CDH2, JAM2, CTLA4, HLA-DRB4, CDH15, CLDN18, CD4, HLA-DRB5, CNTN1, NLGN2, HLA-DRB3, NRXN3, ALCAM, SELE, CD8A, CD8B, CD6, NLGN4X, CLDN17, L1CAM, ITGB8, MAG, VCAN, HLA-F, NFASC, HLA-E, NRXN1, HLA-DPA1, HLA-DPB1, CLDN9, GLG1, HLA-DQA1, HLA-DQA2, HLA-DQB1, NRXN2, CD2, CLDN16, CLDN23, MADCAM1, SDC2, SDC4, CLDN14, CD40, SDC1, OCLN, PVR, HLA-DRB1, NECTIN2, CDH1, HLA-DRA, NECTIN1, HLA-DOA, HLA-DOB, CLDN10, CNTNAP1, ICAM2, ICAM3, CLDN8, CLDN2, CLDN6, CLDN5, CLDN1, ICAM1, NEO1, HLA-C, HLA-B, ESAM, CD40LG, PTPRM, HLA-DMB, HLA-DMA, HLA-A, F11R, PDCD1, CLDN19, PTPRF, CLDN15, CD226, CD99, CLDN22, CNTN2, ITGA6, CNTNAP2, MPZ, MPZL1, PTPRC, NECTIN3, ITGA8, NCAM2, NCAM1, CD58, NEGR1, CLDN11, SDC3, CLDN3, CLDN7, CLDN4	KEGG hsa04514
Complement and coagulation cascades	F2, F2R, VWF, KNG1, FGB, PLAT, SERPIND1, MBL2, F3, F5, SERPINA1, PLAUR, F7, PLAU, F10, F9, C1S, TFPI, F8, KLKB1, CR2, MASP1, C9, A2M, CR1, F12, F13A1, F11, CFI, SERPING1, MASP2, THBD, C1QC, C8A, F13B, C7, C8G, C8B, CD59, SERPINA5, FGG, CD55, C6, C5AR1, C5, BDKRB1, CFD, C1QA, C1R, C4BPB, C4BPA, C4B, C4A, BDKRB2, CFH, CFB, CPB2, CD46, PROS1, SERPINF2, PROC, C3, C1QB, C3AR1, FGA, SERPINE1, PLG, C2, SERPINC1	KEGG hsa04610
TGF-beta signaling pathway	TFDP1, NOG, TNF, GDF7, INHBB, INHBC, COMP, INHBA, THBS4, RHOA, CREBBP, ROCK1, INHBE, THBS2, DCN, ID1, ID2, RPS6KB1, RPS6KB2, CUL1, ID4, SMAD3, MAPK3, RBL2, SMAD4, RBL1, NODAL, THBS1, THBS3, SP1, SMAD1, MYC, SMAD2, MAPK1, SMURF2, SMURF1, EP300, BMP8A, GDF5, SKP1, CHRD, ZFYVE16, BMP6, BMP5, E2F4, TGFB2, TGFB1, IFNG, CDKN2B, PPP2CB, PPP2CA, PPP2R1A, ID3, SMAD5, RBX1, FST, PITX2, ZFYVE9, BMP7, PPP2R1B, TGFBR2, AMHR2, LTBP1, LEFTY1, AMH, TGFBR1, SMAD9, LEFTY2, SMAD7, ROCK2, BMP8B, ACVR1C, TGFB3, SMAD6, BMPR2, GDF6, BMPR1A, BMPR1B, ACVRL1, ACVR2B, ACVR2A, ACVR1, BMP4, E2F5, BMP2	KEGG hsa04530
Base excision repair	NEIL2, MPG, SMUG1, XRCC1, POLE4, HMGB1, POLE3, POLD4, MBD4, OGG1, UNG, POLD3, MUTYH, PARP1, LIG1, PCNA, NEIL1, POLE2, PARP4, PARP3, PARP2, POLB, APEX1, POLL, POLD1, POLD2, POLE, NEIL3, FEN1, TDG, APEX2, LIG3, HMGB1P1, NTHL1, HMGB1P40	KEGG hsa03410
DNA replication	DNA2, POLE4, POLE3, PRIM1, PRIM2, POLD4, RFC4, RFC5, RPA1, POLA1, RPA3, POLD3, LIG1, SSBP1, FEN1, RNASEH2B, RPA2, PCNA, RPA4, RNASEH1, RNASEH2C, MCM4, POLE2, MCM3, MCM6, MCM5, POLA2, FEN1, MCM2, MCM7, POLD1, POLD2, RNASEH2A, POLE, RFC1, RFC3, RFC2	KEGG hsa03030
Nucleotide excision repair	MNAT1, POLE4, ERCC4, POLE3, ERCC3, ERCC6, ERCC5, GTF2H5, POLD4, ERCC2, RFC4, CETN2, RFC5, GTF2H3, RPA1, RAD23B, RBX1, DDB2, RPA3, POLD3, RPA2, RAD23A, PCNA, RPA4, DDB1, POLE2, ERCC1, POLD1, GTF2H4, POLD2, POLE, RFC1, RFC3, RFC2, XPC, XPA, GTF2H2, GTF2H1, CDK7, LIG1, CUL4A, CUL4B, ERCC8,CCNH	KEGG hsa03420
Mismatch repair	MLH3, POLD1, MLH1, POLD2, RFC1, MSH2, RFC3, RFC2, MSH3, POLD4, PMS2, RFC4, LIG1, SSBP1, RPA4, EXO1, RFC5, RPA1, MSH6, RPA3, POLD3, RPA2, PCNA	KEGG hsa03430

**Table 2:** Univariate analysis of the hazard ratio with 95% confidence interval of each gene.

Gene	Hazard ratio	HR.95%L	HR.95%H	*P* value	Gene	Hazard ratio	HR.95%L	HR.95%H	*P* value
AGRN	0.980	0.814	1.181	0.8352	ITGA8	1.101	0.939	1.292	0.2352
CD36	1.344	1.133	1.595	0.0007	ITGA9	1.158	0.985	1.362	0.0753
CD44	1.134	0.982	1.310	0.0864	ITGAV	1.405	1.112	1.777	0.0044
CD47	0.824	0.626	1.086	0.1689	ITGB1	1.192	0.941	1.510	0.1450
CHAD	0.997	0.785	1.267	0.9793	ITGB3	1.286	0.991	1.669	0.0587
COL11A1	1.115	0.987	1.260	0.0795	ITGB4	0.870	0.762	0.995	0.0418
COL11A2	0.843	0.679	1.046	0.1212	ITGB5	1.329	1.030	1.715	0.0287
COL1A1	1.138	1.017	1.273	0.0243	ITGB6	1.102	0.971	1.251	0.1315
COL1A2	1.168	1.033	1.322	0.0135	ITGB7	1.040	0.810	1.334	0.7596
COL2A1	1.089	0.960	1.234	0.1850	ITGB8	0.995	0.813	1.219	0.9635
COL3A1	1.173	1.041	1.321	0.0087	LAMA1	1.044	0.830	1.312	0.7142
COL4A1	1.251	1.042	1.502	0.0164	LAMA2	1.288	1.085	1.529	0.0038
COL4A2	1.196	1.006	1.421	0.0427	LAMA3	1.033	0.902	1.183	0.6366
COL4A4	1.118	0.911	1.373	0.2860	LAMA4	1.298	1.080	1.560	0.0054
COL4A6	1.134	0.916	1.404	0.2471	LAMA5	0.937	0.790	1.112	0.4558
COL5A1	1.163	1.013	1.335	0.0319	LAMB1	1.244	1.014	1.526	0.0363
COL5A2	1.233	1.060	1.433	0.0065	LAMB2	0.999	0.814	1.227	0.9948
COL5A3	1.104	0.909	1.340	0.3173	LAMB3	0.987	0.859	1.133	0.8490
COL6A1	1.135	0.972	1.326	0.1091	LAMB4	2.320	0.762	7.066	0.1386
COL6A2	1.170	1.014	1.349	0.0312	LAMC1	1.277	1.064	1.532	0.0086
COL6A3	1.162	1.013	1.333	0.0319	LAMC2	1.056	0.936	1.191	0.3788
COL6A6	1.361	0.661	2.802	0.4030	LAMC3	0.954	0.741	1.226	0.7113
COMP	1.040	0.949	1.139	0.3989	RELN	1.183	0.976	1.435	0.0874
DAG1	0.985	0.787	1.234	0.8958	SDC1	0.951	0.823	1.099	0.4968
FN1	1.144	1.028	1.273	0.0139	SDC2	1.381	1.144	1.667	0.0008
GP1BA	1.026	0.772	1.364	0.8576	SDC3	0.904	0.740	1.103	0.3203
GP5	1.652	0.676	4.035	0.2708	SDC4	1.068	0.903	1.265	0.4417
GP6	1.187	0.603	2.338	0.6198	SPP1	1.047	0.962	1.139	0.2848
GP9	1.862	0.666	5.210	0.2362	SV2A	1.205	0.967	1.502	0.0962
HMMR	0.917	0.756	1.113	0.3811	SV2B	2.033	1.269	3.257	0.0032
HSPG2	1.087	0.929	1.272	0.2990	SV2C	1.144	0.433	3.027	0.7859
IBSP	1.182	0.957	1.461	0.1212	THBS1	1.210	1.069	1.369	0.0025
ITGA1	1.193	1.001	1.421	0.0484	THBS2	1.138	1.023	1.265	0.0171
ITGA10	1.439	0.958	2.161	0.0792	THBS3	1.220	0.936	1.590	0.1417
ITGA11	1.163	0.994	1.361	0.0602	THBS4	1.058	0.979	1.144	0.1515
ITGA2	0.989	0.837	1.168	0.8945	TNC	1.082	0.978	1.197	0.1245
ITGA2B	1.801	0.666	4.876	0.2466	TNN	1.252	1.020	1.536	0.0314
ITGA3	1.069	0.900	1.270	0.4475	TNR	1.436	0.616	3.349	0.4024
ITGA4	1.175	0.953	1.449	0.1318	TNXB	1.102	0.964	1.261	0.1549
ITGA5	1.129	0.977	1.306	0.1006	VTN	1.134	1.041	1.235	0.0040
ITGA6	0.876	0.729	1.052	0.1561	VWF	1.285	1.085	1.521	0.0036
ITGA7	1.041	0.901	1.202	0.5864					

## Data Availability

The datasets used and/or analyzed during the current study are available from the corresponding author on reasonable request.

## Abbreviations

ECM: Extracellular matrix
TCGA: The Cancer Genome Atlas
lasso: Least absolute shrinkage and selection operator method
STAD: Stomach adenocarcinoma
FPKM: Fragments per kilobase million
KEGG: Kyoto Encyclopedia of Genes and Genomes
CAMs: Cell adhesion molecules
GSEA: Gene set enrichment analysis
ssGSEA: Single-sample gene set enrichment analysis
CAC: Colon adenocarcinoma
OS: Overall survival
BP: Biological process
MF: Molecular function
CC: Cellular component
HR: Hazard ratios
95% CI: 95% confidence interval
logFC: log_2_(fold change)
PCA: Principal component analysis.

## Supplementary Materials

Supplementary materials Supplementary Figure 1: paired *t*-test of ECM-receptor interaction-related genes with statistical difference of gastric cancer in the training cohort (TCGA). Supplementary Figure 2: paired *t*-test of ECM-receptor interaction-related genes in the validation cohort (GSE29272). Supplementary Figure 3: consensus matrixes of the training cohort for *k* from 3 to 9. Supplementary Table 1: patients' clinical characteristics and their cluster classification.
